# The Role of Exosomes in the Pathophysiology of Chronic Rhinosinusitis

**DOI:** 10.3389/fcimb.2021.812920

**Published:** 2022-01-28

**Authors:** Sarina K. Mueller

**Affiliations:** Department of Otolaryngology, Head and Neck Surgery, Friedrich-Alexander-Universität Erlangen-Nürnberg (FAU), Erlangen, Germany

**Keywords:** chronic rhinosinusitis (CRS), exosome, cystatin, pappalysin-1, cell communication

## Abstract

Non-invasive biomarker analysis has made repetitive and painless sampling over time possible. Exosomes are being released from a parent cell and their cargo mirrors the cell micromilieu of the parent cell. Therefore, exosomes are promising surrogates for their parent cells. That is also why exosomes provide an improved signal-to-noise ratio. Current studies have identified valid non-invasive biomarkers that may be able to monitor disease severity. Exosomes are suggested to play an important role in interepithelial communication and are suggested to play a role in the initiation and maintenance of inflammation in CRS. They are, however, also involved simultaneously in several immunological processes including immune protection and immunosuppression. As the isolation of exosomes is time-consuming their value in everyday routine diagnostics has yet to be determined.

## Introduction

Chronic rhinosinusitis (CRS) is one of the most common diseases worldwide and leads to a high socioeconomical burden. The annual direct costs (medication, surgery) or indirect costs (days off, reduced labor productivity) add up to 20 billion US$ (USA 2017) (Rudmik, 2017). CRS patients report the subjective reduction in their quality of life as being worse than patients with chronic heart failure or chronic obstructive pulmonary disease (COPD) (Metson and Gliklich, 2000; Soler et al., 2011). Diagnosis is based on the ICAR : RS (Orlandi et al., 2021) criteria. The existence of a type 2 response, including tissue/serum eosinophils, tissue/serum IgE and the cytokines Il-4, Il-5 and Il-13, is currently the only measure for classifying the disease and predicting potential therapies (Fokkens et al., 2020). The identification and validation of specific and sensitive biomarkers that can diagnose different subtypes of the disease and predict its course are a main priority. Biomarkers can be analyzed in tissue, blood, and nasal secretions (Maxfield et al., 2018; Khan et al., 2019; Mueller et al., 2019a). Tissue biomarkers reflect local nasal inflammatory processes and the nasal microenvironment most closely. However, tissue biopsies from sinus mucosa can only be sampled invasively and at the time point of surgery. Presurgical or longitudinal sampling is not possible. This is why non-invasive mediums, including peripheral blood and nasal secretions, have been studied further. This means that painless and repetitive sampling over time and therefore longitudinal follow-up is possible (Massey et al., 2020). However, peripheral blood is a highly systemic medium representing pathophysiological processes of the whole body. Nasal mucus or nasal lavage fluid are easiest to sample and are accompanied by the lowest side effects. However, the respiratory mucosa is part of the first line of defense, and nasal secretions are therefore in contact with a multitude of pathogens. This is why the question of an improved signal-to-noise ratio for peripheral blood and nasal secretions arose. One answer for improving the signal-to-noise ratio is the use of exosomes (Mueller, 2018; Mueller et al., 2018; Mueller et al., 2019a). Exosomes are spherical to cup-shaped, 30-150nm (Simons and Raposo, 2009; Raposo and Stoorvogel, 2013) vesicles which are secreted by virtually all cell types into a range of body fluids including nasal mucus (Simons and Raposo, 2009; Raposo and Stoorvogel, 2013). Exosomes incorporate proteins, lipids, DNA, miRNA and RNA that are specific to their cell of origin. Consequently, they can be used as a biomarker for their cell of origin (Pisitkun et al., 2006; Valadi et al., 2007; Qin and Xu, 2014). Due to their ubiquitous presence and easy accessibility, exosomes show great potential for non-invasive diagnostics and conducting “liquid biopsies” (Crowley et al., 2013).

Therefore, the objective of this review was to analyze the current literature concerning the role of exosomes in the pathophysiology of CRS.

## Advantages and Disadvantages in the Usage of Exosomes

Exosomes are cell specific and reflect cell functions and conditions. This is why they show great potential as novel biomarkers for clinical diagnosis. Furthermore, exosomes protect their cargo from degradation by nucleases and proteases, thereby increasing the biomarker half-life. Exosomes resemble the tissue proteome more closely than whole mucus and show an improved signal-to-noise ratio (Mueller et al., 2019a). However, the handling of exosomes is not trivial. The majority of current techniques of exosomes processing only concentrates the exosomes and does not isolate them. Consequently, there may be chance of incorrect conclusions regarding biological activity and function of exosomes (Mueller et al., 2018). The most common technique for exosome concentration is ultracentrifugation procedure (Théry et al., 2006; Nocera et al., 2017). In a sequence of different centrifugation steps, exosomes may be successfully concentrated along with other particles with the same size and buoyant density. Ultracentrifugation is time consuming and requires costly equipment. Therefore, the application of exosomes in point-of-care service may be difficult (Mueller et al., 2018). A technique that isolates the exosomes is based on immunoaffinity-based isolation. This technique uses antibody-coated magnetic beads and results in an improved output compared to ultracentrifugation. However, only exosomes containing the target protein can be isolated with that method (Tauro et al., 2012). Another frequently used method is the use of commercial precipitation kits. This method is probably the easiest to implement into clinical practice, however, the purity is reported to be lower as compared to ultracentrifugation techniques using a sucrose density gradient (Van Deun et al., 2014) Further isolation and concentration techniques are summarized in a specific review about exosomes in aerodigestive mucosa (Mueller et al., 2018).

## Potential Non-Invasive Exosomal Biomarkers

Several promising biomarkers have been analyzed in exosomes sampled from CRS patients. Those biomarkers include proteins (Mueller et al., 2018; Miyake MM et al., 2019; Mueller et al., 2019b; Mueller et al., 2019; Mueller et al., 2020; Shin et al., 2020) and miRNA (Shin et al., 2020; Zhang et al., 2020). **Table 1** summarizes all exosomal biomarkers including their physiological function and their supposed role in CRS.

**Table 1:** Summary of all exosomal biomarkers including their physiological function and their supposed role in CRS.

Study	Year published	Biomarker	Physiologic function	role in CRS
Lässer et al., 2016	2016	Nitric oxide	endothelium-derived relaxing factor (EDRF), e.g. vasodilation	immune defense in respiratory tract
Nocera et al., 2017	2017	p-glycoprotein (P-gp)	ATP-binding cassette, anti-efflux membrane pump	immunomodulatory role in Th2 inflammation
Mueller et al., 2018	2018	serpins	serine protease inhibitors, coagulation and fibrinolysis cascade	coagulation and fibrinolysis cascade
Mueller et al., 2019b	2019	serpins	serine protease inhibitors, coagulation and fibrinolysis cascade	coagulation and fibrinolysis cascade
Nocera et al., 2019	2019	Nitric oxide	endothelium-derived relaxing factor (EDRF), e.g. vasodilation	immune defense, innate immunosurveillance
Miyake MM et al., 2019	2019	cystatin-SA (CST-2)	cysteine protease inhibitor	segregation of phenotypes, disease severity monitoring
Zhang et al., 2020	2020	miRNA-22 (MiR-22)	microRNA, mediation of inhibition of translation	enhancement of tubular permeability
Shin et al., 2020	2020	miRNA-19A/miRNA-61	microRNA, mediation of inhibition of translation	promotion of proinflammatory macrophage polarization
Mueller et al., 2020	2020	pappalysin-1 (PAPP-A)	metalloendopeptidase/secreted protease, growth promotion	early recurrence prediciton, disease severity monitoring
Wang et al., 2021	2021	MUC-5A	glycoprotein, first line of defense in respiratory tract	induction of proinflammatory proteins

### Pathophysiological Roles of Exosomes in CRS

#### Role in Interepithelial Transfer

Intercellular communication is the transfer of cargo between an originating cell and a target cell. The traditional ways of intercellular communication are autocrine, paracrine and endocrine or by cell-to-cell-contacts (juxtacrine interaction) (Singh AB, 2005). However, a more sophisticated way of communication includes the exchange of information through the release of membrane-bound bodies called extracellular vesicles (Al-Nedawi et al., 2009). Extracellular vesicles including exosomes can transfer their cargo between close but also between distant cells by travelling through body fluids (Peinado et al., 2012). Therefore, the mechanism of transfer by extracellular vesicles may be interesting for various pathological states including the spreading of tumor cells or inflammatory markers (Peinado et al., 2012; Bleier et al., 2013). For CRS, exosomes may be sampled either from nasal mucus (Mueller et al., 2018; Miyake M et al., 2019b; Mueller et al., 2019c; Mueller et al., 2019; Mueller et al., 2020; Shin et al., 2020), nasal lavage fluid (Choi et al., 2014; Lässer et al., 2016; Zhang et al., 2020; Zhou et al., 2020; Wang et al., 2021) or tissue (Shenoy et al., 2020). In order to analyze the capability of exosomes to transfer cargo from cell to cell, *in vitro* and *in vivo* transfection experiments may be performed. *In vitro* exosomes may play a role in the initiation of inflammation after exposure with external stimuli e.g. air pollutants (Shin et al., 2020). Additionally, exosomes may play an even larger role in maintaining the inflammation in CRS by circling proinflammatory cargo from one cell to another (Bleier, 2012). Hereby, exosomes cannot only transfer cargo between autologous cells but also between epithelial cells of different anatomical regions (Zhang et al., 2020). Most importantly for the maintenance of inflammation, exosomes may transfer their cargo from a disease cell to a non-diseased (control). In the following, characteristic differentially expressed proteins measured to be upregulated in the diseased cell but not in the control cell, are found to also be overexpressed in the control cell after transfection (Zhou et al., 2020; Wang et al., 2021). It is suspected that the interepithelial transfer is similar *in vivo (* Nocera et al., 2019).

#### Role in Immune Response

For CRS, several endotypes have been described for CRSwNP and CRSsNP (Tomassen et al., 2016). Comparable to immune responses in the lower airways, Th1- and Th2- responses have also been described for CRS. Originally, CRSwNP was associated with a Th2- based endotype whereas CRSsNP was associated with a Th1-response. However, the complexity of the immune response may be much greater than expected with different combinations of immune responses in one phenotype (Delemarre et al., 2020; Delemarre et al., 2021). It is suggested that mRNAs or proteins that play a role in inflammatory immune mechanisms may be transferred by exosomes (Wang et al., 2021). Therefore, inflammation may be spread between epithelial cells. Simultaneously, exosomes may also contain information for fighting inflammatory processes and carry immune protective content (Lässer et al., 2016; Nocera et al., 2019). In this context, exosomes may attract immune cells of pathological epithelial cells from CRS or asthma patients. A migration of monocytes, neutrophils and NK cells have been described *in vitro* (Lässer et al., 2016). When analyzing the exosomal proteome, it could be strongly correlated with pro- as well as anti-inflammatory proteins which may be present at the same time (Lässer et al., 2016; Mueller et al., 2019a). One enzyme that was found to be biologically active even within exosomes was the inducible nitric oxide synthase (iNOS) (Lässer et al., 2016; Nocera et al., 2019). Nitric oxide may play an important role in the immune protection of the airway epithelium as a first line of defense. NO can also be induced by lipopolysaccharides (LPS). LPS can be found in the outer membrane of Gram-negative bacteria and are known to stimulate toll-like receptor 4 (TLR-4). It could be shown that LPS lead to a secretion of exosomes into nasal mucus *in vitro* and *in vivo* (Nocera et al., 2019). Those exosomes increased in number after LPS induction and exosomal inducible nitric ocide synthase expression was also increased. It is suggested that the LPS stimulation led to increased exosomal microbiocidal activity against Pseudomonas aeruginosa. Moreover, NO may be transferred between epithelial cells, thus increasing NO production within nasal epithelial cells (Nocera et al., 2019). Interestingly, transfection experiments indicate that the protein transfer was completed within 5 minutes of contact (Nocera et al., 2019). Hence, exosomes may therefore play a role in the innate immunosurveillance and defense mechanism of the human upper airways. Nasal mucus derived exosomes seem to harbor immune defensive proteins, which suggests that exosomes may also play a role in maintaining the barrier function of the sinonasal mucosa.

#### Role in Predicting Disease Severity and Recurrence

Despite CRS being a common disease, the pathophysiology of recurrences as well as the prediction of disease severity remain elusive. Nor has a common definition of recurrence has been established. A classification based on the time point of recurrence or disease severity based on endotypes is desperately needed. Exosomal research on protein biomarkers provides one approach to solving this shortcoming. Nasal mucus derived exosomes may contain cystatin-SA (CST-2), a cysteine protease inhibitor. Exosomal CST-2 was correlated with clinical and demographical variables including allergies, asthma, eosinophilia and non-steroidal anti-inflammatory respiratory disease-exacerbated respiratory disease. It is suggested that CST-2 expression can segregate the phenotype. Additionally, CST-2 levels may cluster CRS patients based on disease severity (Miyake MM et al., 2019). CST-2 may moreover play a part in disease recurrence as do periostin and pappalysin-1. Although the study was performed in whole mucus all protein biomarkers have been shown to also be exosomal (Mueller et al., 2019a; Mueller et al., 2020). All biomarkers decreased after sinus surgery and increased postoperatively. An earlier and steeper increase correlated with earlier recurrences. An increase in the combination of periostin, CST-2 and pappalysin-1 may therefore predict early recurrences in CRS. Interestingly, this combination of biomarkers increased their levels months before the patients experienced symptoms measured with the rhinologic domain of the SNOT-22 score (Mueller et al., 2019c). Those nasal mucus derived proteins may therefore play a role in the diagnosis of exacerbations and the prediction of recurrences.

#### Role in the Coagulation and Fibrinolysis Pathways

The coagulation and fibrinolysis pathways have been studied by several groups with respect to CRS (Takabayashi et al., 2013; Kim et al., 2015). Among all protein pathways analyzed by a multiplexed approach, the coagulation cascade was the most significantly associated with CRSwNP (Mueller et al., 2019b). It is suggested that the coagulation cascade is upregulated whereas the fibrinolysis cascade is downregulated. As a consequence, fibrin accumulates which leads to increased edema and polyp growth. These findings could not only been noted in CRSwNP tissue but also in nasal mucus-derived exosomes from CRSwNP patients (Mueller et al., 2018; Mueller et al., 2019b). As the coagulation and fibrinolysis cascades are complex due to their frequent interaction with other pathways, sufficient substrate is necessary for analysis. Due to the non-invasive and repetitive sampling possibilities, exosomes represent one method that allows frequent sampling of high-quality substrate.

#### Role in Microbiome

“The microbiome is the collection of all microbes, such as bacteria, fungi, viruses and their genes that live naturally on our bodies and inside us” (https://www.niehs.nih.gov/). Regarding external causes to explain the etiology of CRS, different bacteria have been discussed. Interestingly, exosomes may also be released by bacteria and therefore may be an important causative agent of inflammation. The metagenomic analysis of the nasal lavage fluid microbiome showed that the composition of the bacteria was positively correlated with the exosome composition. For bacteria and their exosomes, patients with CRS had greater bacterial abundance and lower diversity compared to controls. Additionally, an increased Staphylococcus aureus abundance was seen in CRSwNP patients (Choi et al., 2014). This mechanism may be one explanation for the etiology of CRS and the initiation of inflammation by exosomal cargo from bacteria.

#### Role Immune Suppression by T Cell Checkpoints

Immune checkpoints are physiologically integrated into the immune system. Their role is to prevent an excessive immune response that destroys healthy cells in the body. At the latest since the Nobel Prize was presented to Tasuku Honjo and James P. Allison in 2018, immune checkpoint inhibitors, including programmed cell death protein-1 (PD-1) or cytotoxic T-lymphocyte-associated Protein 4 (CTLA-4), have been ubiquitously present in the treatment of cancer. Accordingly, research on T cells and their activation patterns has also been carried out in CRSwNP. As already mentioned in previous paragraphs, exosomes may contain immunogenic as well as immunosuppressive content. In this context, tissue exosomes of CRSwNP patients may be able to arrest the activation of T cells stimulated by the T cell receptor. Similarly to the tumor microenvironment, these exosomes can lead to an immune suppression and inhibit T cell activation. In CRSwNP, this tumor suppression may be dependent on ganglioside GD3, which is expressed on the exosomal surface. Consequently, exosomes with immunosuppressive characteristics may represent a T cell checkpoint and therefore a target for novel treatment options (Shenoy et al., 2020).

## Shortcomings and Future Perspectives

Exosomal research is a small but increasing field of activity in rhinology and in studying CRS. The non-invasive sampling through whole nasal mucus or nasal lavage fluid has made painless and repetitive sampling over time possible. The usage of exosomes is beneficial due to the improved signal-to-noise-ratio. Although the application of exosomal biomarkers would be suitable, today, no study has analyzed the potential for the diagnosis or prediction of acute exacerbations. Ideally, an exosomal biomarker would increase before subjective symptoms of an acute exacerbation of CRS (AECRS) emerge. This would give the physician the time to react and if necessary, escalate medical therapy. Future studies in this area are urgently needed. Importantly, a unified definition of AECRS should be used in those studies to guarantee comparability and uniformity. Promising biomarkers of the current literature may be evaluated specifically for AECRS (Mueller et al., 2019c; Wu et al., 2019; Wu et al., 2020).

Exosomes may represent one approach to explain interepithelial communication. However, it is not entirely clear which cargo ultimately ends up in the exosome to be transported to neighboring cells. Additionally, it is not clear how many proteins or mRNAs are needed to be differentially expressed in order to lead to a pathological change in the receiving cell. These questions would be crucial to answer as the transfer of pathological proteins comprises the chance of interfering with novel therapeutic options. Future studies are needed to solve these shortcomings.

## Conclusions

Various exosomal biomarkers have been investigated. Exosomal biomarkers may play an important role in the prediction of the severity of CRS, polyp growth and recurrence, the initiation and maintenance of inflammation, vascular permeability and immune protection as well as immunoinhibition. Several studies have suggested that exosomes play a crucial role in the transport of their cargo from one cell to another, even between different epithelial cell origins. This mechanism may be a key for spreading and maintaining disease and is one step towards the analysis of the pathophysiology of CRS. Research on exosomes allows studying the pathophysiology of CRS as well as prospective biomarkers. However, in this relatively new field, future studies are needed to validate the results.

## Author Contributions

The author confirms being the sole contributor of this work and has approved it for publication.

## Conflict of Interest

The author declares that the research was conducted in the absence of any commercial or financial relationships that could be construed as a potential conflict of interest.

## Publisher’s Note

All claims expressed in this article are solely those of the authors and do not necessarily represent those of their affiliated organizations, or those of the publisher, the editors and the reviewers. Any product that may be evaluated in this article, or claim that may be made by its manufacturer, is not guaranteed or endorsed by the publisher.
